# The Antifungal Effects of Berberine and Its Proposed Mechanism of Action Through CYP51 Inhibition, as Predicted by Molecular Docking and Binding Analysis

**DOI:** 10.3390/molecules29215079

**Published:** 2024-10-27

**Authors:** Chao-Wei Zhang, Dong-Yu Huang, Muhammad Shahid Riaz Rajoka, Yan Wu, Zhen-Dan He, Liang Ye, Yan Wang, Xun Song

**Affiliations:** 1 School of Pharmacy, Shenzhen University Medical School, Shenzhen University, Shenzhen 518055, China; 2110245018@email.szu.edu.com (C.-W.Z.); 18218793343@163.com (D.-Y.H.); shahidrajoka@yahoo.com (M.S.R.R.); liangyeszu@163.com (L.Y.); 2 College of Pharmacy, Shenzhen Technology University, Shenzhen 518118, China; wuyan@sztu.edu.cn (Y.W.); hezhendan@sztu.edu.cn (Z.-D.H.); 3 Center for Translation Medicine Research and Development, Shenzhen Institutes of Advanced Technology, Chinese Academy of Sciences, Shenzhen 518055, China

**Keywords:** berberine, antifungal, *Candida albicans*, *Cryptococcus neoformans*, *Trichophyton*, CYP51 inhibition

## Abstract

Fungal infections present a significant health risk, particularly in immunocompromised individuals. Berberine, a natural isoquinoline alkaloid, has demonstrated broad-spectrum antimicrobial activity, though its antifungal potential and underlying mechanisms against both yeast-like and filamentous fungi are not fully understood. This study investigates the antifungal efficacy of berberine against *Candida albicans*, *Cryptococcus neoformans*, *Trichophyton rubrum*, and *Trichophyton mentagrophytes* in vitro, as well as its therapeutic potential in a murine model of cryptococcal infection. Berberine showed strong antifungal activity, with MIC values ranging from 64 to 128 µg/mL. SEM and TEM analyses revealed that berberine induced notable disruptions to the cell wall and membrane in *C. neoformans*. No signs of cell necrosis or apoptosis were observed in fungal cells treated with 2 × MIC berberine, and it did not increase intracellular ROS levels or affect mitochondrial membrane potential. Molecular docking and binding affinity assays demonstrated a strong interaction between berberine and the fungal enzyme CYP51, with a dissociation constant (KD) of less than 1 × 10^−12^ M, suggesting potent inhibition of ergosterol biosynthesis. In vivo studies further showed that berberine promoted healing in guinea pigs infected with *T. mentagrophytes*, and in a murine cryptococcal infection model, it prolonged survival and reduced lung inflammation, showing comparable efficacy to fluconazole. These findings indicate that berberine exerts broad-spectrum antifungal effects through membrane disruption and CYP51 inhibition, highlighting its potential as a promising therapeutic option for fungal infections.

## 1. Introduction

Fungal infections caused by pathogenic species such as *Candida albicans*, *Cryptococcus neoformans*, *Trichophyton rubrum*, and *Trichophyton mentagrophytes* are a growing concern in clinical settings, particularly due to their association with immunocompromised patients and the emergence of drug-resistant strains [1,2]. Traditional antifungal agents, such as azoles, target ergosterol biosynthesis by inhibiting lanosterol 14α-demethylase (CYP51), a key enzyme in maintaining fungal cell membrane integrity [2]. However, the development of resistance to azoles, especially fluconazole, has highlighted the urgent need for new antifungal compounds with novel mechanisms of action [3].

Berberine, a natural isoquinoline alkaloid found in various medicinal plants, has gained recognition for its broad-spectrum antimicrobial effects, including potential antifungal activity [4]. This bioactive compound is commonly extracted from the roots, rhizomes, and bark of plants belonging to families like *Ranunculaceae* [5], *Rutaceae* [6], and *Berberidaceae* [7]. Its use in traditional Chinese medicine dates back to approximately 3000 BC, with early references found in the ancient medical text, “The Divine Farmer’s Herb-Root Classic” [8]. Notably, berberine has been used in combination with amphotericin B to treat disseminated candidiasis in murine models [9]. Furthermore, when paired with fluconazole, berberine has shown promise as an effective and safe agent against fluconazole-resistant *Candida albicans* in vitro, offering potential in overcoming drug resistance in fungal infections [10]. In clinical practice, the application of berberine’s antifungal properties holds significant promise, especially amid the growing threat of drug-resistant fungal infections. Berberine has experienced a significant rise in global popularity, driven by its broad therapeutic potential and an expanding body of scientific research. Berberine hydrochloride, widely used as an over-the-counter drug in China, is acknowledged in the “Pharmacopoeia of the People’s Republic of China” as an oral antibacterial treatment. It is commonly prescribed for gastrointestinal infections, with the typical dosage being 0.1 g, taken 1–3 times per day [11]. Initially recognized in traditional Chinese medicine, berberine has gained traction in Western countries for its potential to manage chronic conditions such as type 2 diabetes, high cholesterol, and obesity [12]. Beyond its standalone efficacy, berberine enhances the potency of antibiotics and antifungal agents through synergistic interactions. Notably, it disrupts the biofilm matrix of several pathogens, including multidrug-resistant (MDR) bacteria and fungi, which contributes to its wide-ranging antimicrobial activity [13]. However, the specific mechanisms behind berberine’s antifungal effects are still not fully understood and have been sparsely studied.

This study aims to further investigate the antifungal properties of berberine and involve multiple mechanisms, including fungal morphology, the disruption of fungal cell membranes, interference with mitochondrial function, and its interaction with CYP51. By combining molecular docking, binding affinity assays, and in vitro antifungal evaluations, this research seeks to elucidate the mechanisms underlying berberine’s antifungal activity and its potential as a therapeutic option for combating resistant fungal infections.

## 2. Results

### 2.1. Antifungal Effects of Berberine In Vitro

**Figure 1:**
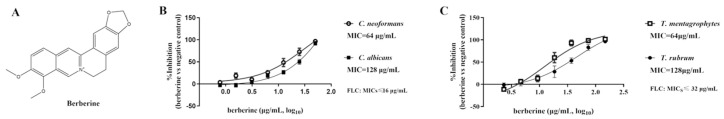
(**A**) Molecular structure of berberine; (**B**) dose−response curve of berberine showing its inhibitory effects on *Candida albicans* (ATCC 10231) and *Cryptococcus neoformans* (ATCC 32609). The antifungal activity of berberine was evaluated using a seven−point dose−response in a microbroth dilution assay. (**C**) Dose−response of *Trichophyton rubrum* (ATCC−MYA4438) and *Trichophyton mentagrophytes* (ATCC−MYA4439) conidia incubated for 72 h with varying berberine concentrations. The results represent two independent experiments, each conducted in triplicate, with data plotted as a percentage of control (100%) for each dose point.

Standard fungal strains from the American Type Culture Collection (ATCC) were chosen to ensure consistency, reliability, and reproducibility in research. The in vitro antifungal activity of berberine, as illustrated in Figure 1, demonstrated its efficacy against both yeast-like fungi and filamentous fungi. Berberine exhibited antifungal effects on *C. albicans* and *C. neoformans*, with 50% inhibition rates ranging from 30.92 to 50.93 µg/mL, and minimum inhibitory concentration (MIC) values between 64 and 128 µg/mL. It also displayed activity against *T. rubrum*, with an MIC value of 128 µg/mL. For the filamentous fungus *T. mentagrophytes*, berberine showed an MIC of 64 µg/mL, highlighting its effectiveness across different fungal species.

### 2.2. Electron Microscopy

**Figure 2:**
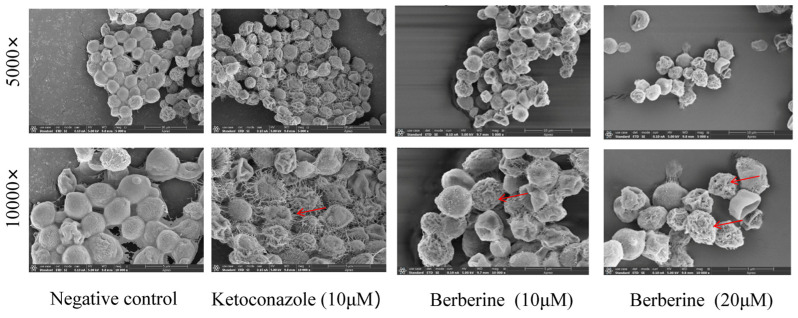
SEM−based surface morphological evaluation of *C. neoformans* cells to assess the effect of 24 h ketoconazole and berberine post−treatment. The presence of holes in the cell surface is indicated by the red arrows. The cells were examined at magnifications of 5000× and 10,000× under SEM. The scale bar for images is 1.0 μm.

Scanning electron microscopy (SEM) analysis was performed to evaluate the effects of berberine on fungal cell morphology and membrane ultrastructure. Untreated *C. neoformans* cells displayed a smooth, intact cell surface (Figure 2). However, exposure to sub−MIC concentrations of berberine resulted in significant alterations to cell surface structure. Damage was clearly visible, including cell wall breakage, surface disruption, and the appearance of holes (highlighted by red arrows), which facilitated the leakage of intracellular contents. Berberine also promoted pore formation on the cell surface, increasing membrane permeability and causing cell deformation. These results indicate that berberine causes extensive disruption and permeabilization of *C. neoformans* cell surfaces, primarily through pore formation.

### 2.3. Ultrastructural Analysis Demonstrated the Effective Activity of Berberine in C. neoformans

**Figure 3:**
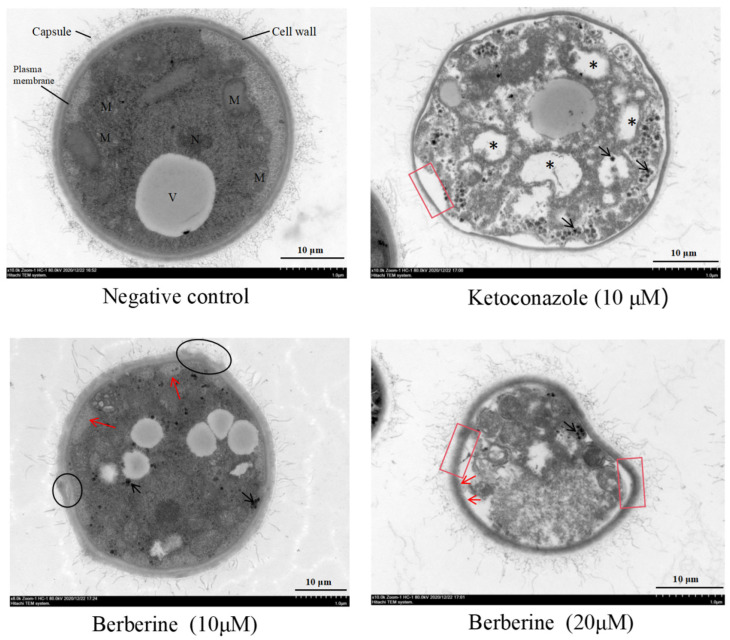
TEM visualization of the cross-sections of the untreated and ketoconazole- and berberine-treated *C. neoformans* cells after 24 h. Cells were observed under TEM at 10,000× magnification. TEM showed the changes in cell ultrastructure after incubation with berberine or ketoconazole, including the following: cytoplasmic vacuoles containing vesicles (black asterisks), intracellular and extracellular vesicles (red arrows), cells showing organelle leakage from vacuoles (black arrows), cell wall breakage (black circles), invaginations of the plasma membrane extending into the cytoplasm, and shrunken cytoplasmic membranes detached from the cell wall (shown in red frames). Three independent experiments, with six biological replicates/group, were performed for each drug treatment. Micrographs in this image represent one of six biological replicates analyzed. M: mitochondrial; V: vacuoles; N: nucleus.

Transmission electron microscopy (TEM) analysis of untreated *Cryptococcus* cells revealed their typical round shape, with smooth contours, uniformly thick cell walls, and a well-defined polysaccharide capsule. Internal organelles appeared intact and well-preserved, with clear visualization of free ribosomes, distinct rough endoplasmic reticulum, multivesicular bodies, vacuoles containing heterogeneous materials of various sizes, and a few lipid droplets (Figure 3). In contrast, cells treated with ketoconazole and berberine (at 10 and 20 μM concentrations) showed markedly altered morphology. A higher incidence of lysed cells and irregular surfaces was observed, corresponding to increasing concentrations of berberine. As shown in Figure 3, control cells retained intact cell walls and membranes, maintaining a conserved structure. However, treatment with ketoconazole or berberine resulted in significant ultrastructural changes, including cytoplasmic membrane shrinkage and detachment from the cell wall (red frame), the formation of bulbous membrane structures, and visible cell wall damage (black circle). Additionally, branched and narrow invaginations extending into the cytoplasm were observed (red arrows). Mitochondrial damage was evident, characterized by disrupted vacuoles, pores, and visible leakage (black arrows). Some organelles, including the nucleus and vacuoles, were no longer discernible, and membrane detachment led to pore formation, resulting in the leakage of intracellular contents. This leakage was more severe in treated cells, eventually causing cell death. These structural changes suggest that berberine compromises the plasma membrane, likely penetrating it and interacting with intracellular organelles.

### 2.4. Berberine Does Not Induce Fungal Necrosis or Apoptosis

**Figure 4:**
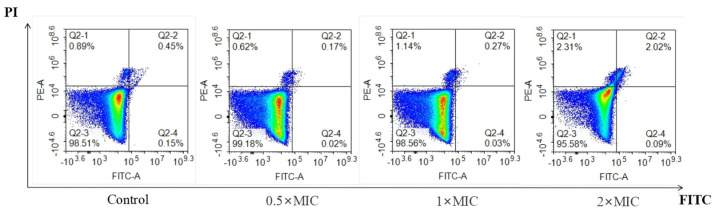
Flow cytometry demonstrated necrotic cell death induced by membrane damage in *C. neoformans* after 4 h of berberine exposure. The study compared negative control cells with those treated with berberine at 0.5 × MIC, 1 × MIC, and 2 × MIC. Q1: necrosis; Q2: late apoptosis; Q3: early apoptosis; and Q4: alive. The x axis (FITC−A) and y axis (PE−A) of the cytometer plot represent the cells’ condition in terms of fluorescent area (A).

Flow cytometric analysis of phosphatidylserine externalization in *C. neoformans* further validated the impact of berberine on cell membrane integrity. As shown in Figure 4, there was no significant increase in PI-stained *C. neoformans* cells after 4 h of berberine treatment (0.62%, 1.14%, and 2.31%) compared to the negative control (0.89%), indicating that berberine did not compromise membrane integrity in these cells.

### 2.5. Evaluation of Reactive Oxygen Species (ROS) Production and Mitochondrial Membrane Potential Dynamics in Response to Berberine Treatment

**Figure 5:**
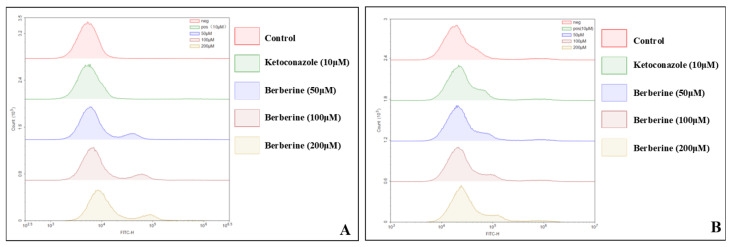
(**A**) Assessment of ROS generation in *C. neoformans* following berberine treatment, as detected by dihydrofluorescein diacetate (DHF) staining; (**B**) analysis of mitochondrial membrane potential alterations in *C. neoformans* post-berberine treatment, using appropriate membrane potential-sensitive dyes.

We used an ROS-sensitive probe, dihydrofluorescein (DHF), which fluoresces upon reacting with free radicals, to assess ROS production in fungal cells. As shown in Figure 5, berberine treatment did not significantly impact intracellular ROS levels. To further explore berberine’s effects on mitochondrial activity, we examined mitochondrial membrane potential, a critical marker of mitochondrial function. Employing the fluorescent dye Rhodamine 123, which accumulates in mitochondria in a membrane potential-dependent manner, we observed no significant increase in Rhodamine 123 accumulation in cells cultured in inducing medium compared to those cultured in Sabouraud medium (Figure 5). These results indicate that berberine does not enhance mitochondrial membrane potential.

### 2.6. Molecular Docking Analysis and Molecular Interaction Analysis

**Figure 6:**
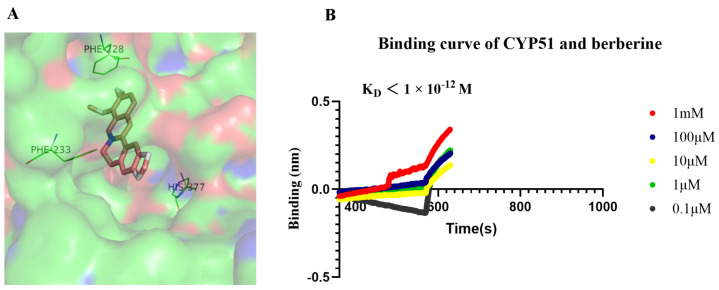
(**A**) Docking of berberine binding to CYP51 Ca (*C. albicans*). Stereoscopic view of CYP51 Ca with berberine bound in active site; (**B**) binding affinity measurement using BLI. The binding affinity of HR1MFd to HR2 was determined by BLI. The fitting curve was analyzed by ForteBio Data Analysis 12.0 software.

Docking studies revealed that berberine binds in close proximity to the active site pocket of *C. albicans* CYP51 (CYP51Ca, binding affinity: −10.6 kcal/mol). The residues surrounding berberine are predominantly hydrophobic, notably Phe228. Berberine’s two methoxy groups and benzene ring form hydrophobic interactions with the Phe228 residue of CYP51. In our model, the distal phenyl ring of berberine, featuring methoxy groups, forms aromatic interactions with His377, while a hydrogen bond is established between the nitrogen atom of berberine and the hydroxyl group of Phe233. To further investigate, we assessed the binding affinity of berberine to CYP51Ca using biolayer interferometry (BLI). As shown in Figure 6, berberine binds to CYP51Ca in a dose-dependent manner, with a dissociation constant (KD) of less than 1 × 10^−12^ M. These findings indicate that berberine exhibits a strong binding affinity to CYP51Ca, which likely contributes to its antifungal properties.

### 2.7. Efficacy of Once-Daily Berberine Application on Treating Dermatophytosis

**Figure 7:**
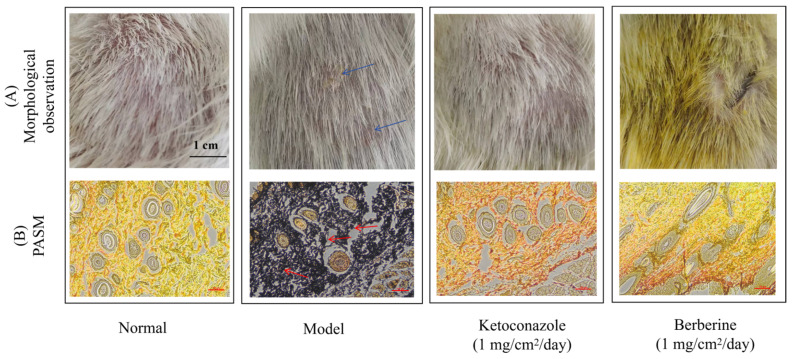
(**A**) Visual appearances of the inoculation sites on guinea pigs across various treatment groups; (**B**) skin tissue samples from guinea pigs under different treatments, highlighting the inflammatory response and epidermal thickness. Staining was performed using periodic-acid silver methenamine (PASM), observed at 100× magnification, with a scale bar representing 100 μm. In the model group, infected skin displayed scabs (blue arrows), and numerous fungal cells, stained black (red arrows), were evident in the back skin of the infected animals.

The effectiveness of once-daily berberine application for treating dermatophytosis was assessed in guinea pigs infected with *T. mentagrophytes*. By the 10th day post-infection, clinical symptoms such as edema, erythema, and mild hair loss became increasingly evident. Infected skin areas developed crusting, reflecting inflammation and tissue damage, along with annular or circular erythematous patches with slightly elevated borders and fine scales, or brownish lesions with similar scaling. On Day 21, the severity of skin lesions varied across treatment groups, as depicted in Figure 7. While the uninfected negative control group displayed normal skin and hair growth (Figure 7A), the untreated model group showed prominent brown patches (Figure 7B). Both the ketoconazole and berberine treatment groups (1 mg/cm^2^/day) demonstrated normal hair regrowth and no visible signs of infection (Figure 7).

Histological analysis revealed normal dermal structure in the negative control group. In contrast, the untreated model group exhibited widespread fungal mycelium, highlighted by PASM staining. However, in the ketoconazole- and berberine-treated groups, fungal hyphae and spores were rarely observed, indicating the efficacy of both treatments.

### 2.8. Administration of Berberine Prolonged Survival and Enhanced Body Weight Following C. neoformans Infection

**Figure 8:**
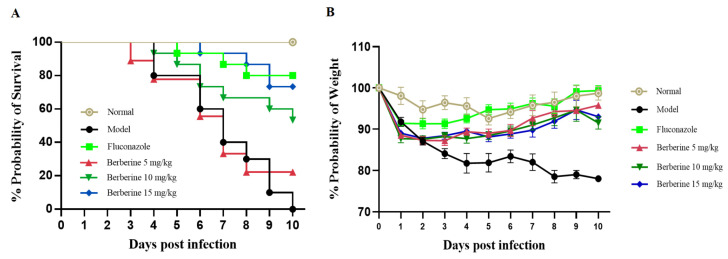
Survival (**A**) and body weight changes (**B**) of immunocompromised mice following infection with *C. neoformans* at a concentration of 1 × 10^8^ CFU/mL. Mice were treated for 10 days with either water (normal control and infection model groups), fluconazole (FLC, 5 mg/kg/day), or berberine at doses of 5, 10, or 15 mg/kg/day.

The in vivo therapeutic efficacy of berberine was evaluated using a cyclophosphamide (CY)-induced immunosuppression murine model of cryptococcal infection, following the method described by Hu *et al.* [14]. As shown in Figure 8, all sham-treated mice receiving sterile water succumbed to the infection by Day 10. In contrast, the group treated with 5 mg/kg of fluconazole (FLC) demonstrated an 80% survival rate within this period. Treatment with 5 mg/kg of berberine improved survival to approximately 70%, while mice administered 10 mg/kg of berberine had a 50% survival rate by Day 10. Additionally, *C. neoformans* infection caused a 20% reduction in body weight over 10 days. Mice treated with berberine began to gain weight rapidly starting on Day 5, with overall weight loss by Day 10 being less than 10%, as shown in Figure 8B, compared to controls.

### 2.9. Efficacy of Berberine in a Murine Model of Cryptococcal Infection

**Figure 9:**
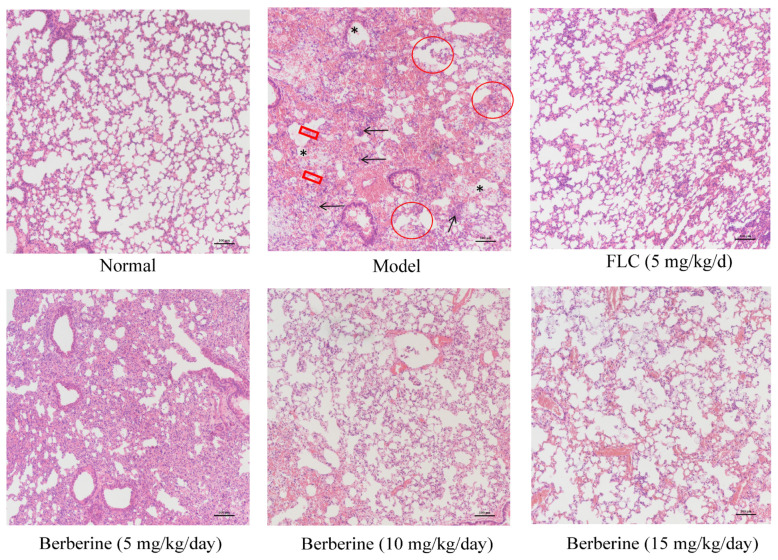
Lung Histopathology. Lung tissue sections were stained with H&E and analyzed from the following groups: normal mice, mice infected with *C. neoformans* alone, and mice treated with 5 mg/kg/day of FLC, 5 mg/kg/day of berberine, 10 mg/kg/day of berberine, and 15 mg/kg/day of berberine. Features observed included widening of the alveolar intervals and thickening of the alveolar septa (red frame), collapsed pulmonary alveoli (red circle), infiltration of inflammatory cells (black arrows), alveolar edema (black star), and structural disorganization. Scale bar = 100 μm.

We assessed lung inflammation using hematoxylin and eosin (H&E) staining. Following euthanasia, lung tissues were collected, fixed in formalin, and embedded in paraffin. Sections were then cut and stained with H&E to visualize histopathological changes. We evaluated inflammation by examining the infiltration of inflammatory cells and the structural integrity of lung tissue under a light microscope. As shown in Figure 9, representative lung images stained with H&E show that uninfected mice maintained a well-structured, normal lung architecture, whereas water-sham-treated mice exhibited significant pathological changes. These included widened alveolar intervals and thickened alveolar septa (red frame), collapsed alveoli (red circle), infiltration of inflammatory cells (black arrows), alveolar edema (black star), and general structural disorganization (Figure 9). In the infection model group, H&E staining revealed severe tissue damage, characterized by extensive inflammatory cell infiltration, including neutrophils, and purulent inflammation, potentially forming small abscesses (black arrows). In contrast, such pathological abnormalities were notably absent in the berberine and FLC treatment groups (Figure 9). These findings suggest that berberine effectively reduces fungal invasion and mitigates tissue damage in infected organs.

## 3. Discussion

Fungal infections pose a significant global health risk, leading to over 180,000 deaths annually from invasive infections [15]. Without treatment, the mortality rate for these infections can reach 100% [15]. Despite these alarming figures, the treatment arsenal for fungal infection remains limited, with only three major drug classes approved for clinical use [16]. Compounding this issue is the limited efficacy of the newest drug class, echinocandins, against *Cryptococcus* species, as well as growing concerns over host toxicity and the development of pathogen resistance with existing treatments [17]. Given these challenges, plant-derived bioactive secondary metabolites are emerging as promising alternatives for antifungal drug development, with ethnobotanical medicines offering valuable leads [18].

In this study, berberine exhibited potent antifungal activity against both yeast-like fungi and filamentous species. These findings align with previous research highlighting berberine’s broad-spectrum antimicrobial properties, particularly its effectiveness against *Candida* species and other opportunistic pathogens [10,19]. For example, Chinese studies have focused on berberine’s role in modulating gut microbiota and its therapeutic potential in treating metabolic disorders [20]. Huang *et al.* further demonstrated berberine’s ability to disrupt fungal cell wall and membrane integrity, contributing to its antifungal effects [21], including inhibiting *C. albicans* biofilm formation [22]. Our findings build on these results by showing that berberine causes significant damage to *C. neoformans* cell walls and membranes, as confirmed by SEM and TEM analyses. Additionally, our study is among the first to report berberine’s antifungal activity against filamentous fungi, particularly dermatophytes like *T. rubrum* and *T. mentagrophytes*. Unlike most earlier studies focusing on yeasts, we demonstrate, for the first time, berberine’s potent activity against *T. mentagrophytes* in vivo, suggesting its potential as a treatment for dermatophytic infections that often resist conventional antifungal therapies.

Morphological analysis revealed that berberine treatment led to pronounced alterations in planktonic fungal cells, including cell disruption and changes in plasma membrane alignment (Figure 2 and Figure 3). The presence of holes in the membrane and a shrunken cytoplasmic membrane indicate that berberine may compromise the integrity of the fungal cell membrane, leading to increased permeability. This effect is likely due to the displacement of ergosterol, which disrupts the lipid bilayer structure and causes leakage of intracellular contents, further supporting our observations. These results are the first to suggest that berberine may cause membrane damage in *C. neoformans*. Further analysis confirmed that treatment with berberine induced significant changes to the cell wall and membrane, as observed via SEM and TEM. Our findings show that berberine causes severe alterations to the fungal cell wall, leading to surface roughness, wrinkling, membrane rupture, and eventual cell collapse. To confirm that berberine-induced membrane damage, we utilized the fluorescent probe PI, which binds to DNA upon entering the cytoplasm [23]. However, flow cytometry analysis showed no significant increase in PI signals in *C. neoformans* (Figure 4), indicating that berberine does not induce apoptosis or necrosis.

Reactive oxygen species (ROS) are known to cause lipid peroxidation and damage organelles, including mitochondria, which can trigger apoptosis and impair fungal cell growth [24]. Mitochondria play a critical role in oxidative phosphorylation, and reduced mitochondrial membrane potential (MMP) is a key indicator of oxidative stress-induced apoptosis [25]. Mitochondria play an important role in maintaining the balance of oxidative phosphorylation, and the reduction in MMP induced by oxidative stress is a late and landmark event in the cell apoptosis pathway [26]. Contrary to previous reports, we found that berberine neither induces ROS accumulation nor decreases MMP (Figure 5). This suggests that berberine inhibits fungal growth through mechanisms independent of ROS production.

Through virtual screening, we identified CYP51, a key enzyme in the ergosterol biosynthesis pathway, as a potential target of berberine. CYP51, a member of the cytochrome P450 superfamily, plays a crucial role in fungal membrane integrity [27]. Inhibition of CYP51 disrupts ergosterol production, compromising membrane structure and leading to increased permeability and cell death [28,29]. Our molecular docking studies and binding affinity assays with recombinant CYP51 proteins support the hypothesis that berberine exerts antifungal effects by targeting this enzyme (Figure 6). Berberine binds to the same amino acid residues at Phe228 and Phe233 as the native isoquinoline ligands (sanguinarine and chelerythrine) and posaconazole [18]. These findings are consistent with previous research suggesting that berberine destabilizes cell membranes.

In addition to inhibiting CYP51, berberine induced substantial morphological changes in fungal cells, including membrane disruption, pore formation, and leakage of intracellular components, as verified through SEM and TEM analyses. Interestingly, unlike prior studies that attributed berberine’s antifungal action to ROS generation, our data suggest that ROS production is not a significant factor in berberine-induced fungal cell death. This discrepancy may be due to differences in fungal species or experimental conditions.

Given its promising in vitro antifungal activity [13,30,31], berberine’s therapeutic potential warrants further investigation in in vivo models. In a guinea pig model of dermatophytosis, external application of berberine effectively inhibited fungal growth and improved clinical symptoms. Direct specimen tests were negative in berberine-treated guinea pigs, suggesting a clinical cure. PASM staining revealed that while some hyphae persisted, berberine’s therapeutic effects on epidermal repair were comparable to those of ketoconazole. This study is the first to show that berberine-treated animals exhibited lower fungal proliferation and inflammation. Additionally, in a systemic fungal infection mouse model, berberine reduced weight loss and improved survival rates in mice infected with *C. neoformans*. High-dose berberine significantly reduced lung tissue infection and inflammatory cell infiltration, promoting recovery in infected mice.

Berberine’s dual mode of action—disrupting cell membranes and inhibiting enzyme activity—makes it a strong candidate for treating fungal infections, particularly those caused by drug-resistant strains. Its broad spectrum of activity and ability to target multiple pathways may reduce the risk of resistance development, a significant issue with current antifungal agents like azoles [32,33]. The use of berberine as an antifungal agent presents challenges, mainly due to its higher MIC compared to modern antifungals, which may limit its effectiveness as a standalone treatment. However, berberine’s real potential lies in its synergistic effects when combined with existing antifungal drugs like fluconazole or amphotericin B. Further exploration of these combinations could enhance efficacy and broaden treatment options for resistant fungal infections [34,35], as combination therapy could enhance efficacy against resistant strains while lowering the required dosages, thus reducing side effects [36,37]. Studies have shown that berberine exhibits synergistic effects when combined with other antimicrobial agents, suggesting this approach may be beneficial in antifungal therapy as well [38,39,40]. Based on the research background and the findings of this study, berberine shows promising potential for development as an in vitro agent or a synergistic adjuvant in the treatment of fungal infections.

## 4. Materials and Methods

### 4.1. Reagents

Berberine, ketoconazole, fluconazole, and cyclophosphamide (CY) each with a purity of over 99%, were purchased from Dalian Meilun Biotechnology Co., Ltd. (Dalian, China). The ROS detection kit and Rhodamine 123 were purchased from Beyotime Biotechnology Co., Ltd. (Shanghai, China). Recombinant protein CYP51 of *C. albicans* was obtained from Sangon Biotech (Shanghai) Co., Ltd. (Shanghai, China).

### 4.2. Pathogens and Culture Conditions

The fungal strains used in the bioassays included *Candida albicans* (ATCC 10231), *Cryptococcus neoformans* (ATCC 66031), *Trichophyton rubrum* (ATCC MYA-4438), and *Trichophyton mentagrophytes* (ATCC 28146). *C. albicans* was cultured at 30 °C in yeast malt (YM) medium (BD Biosciences, San Jose, CA, USA), while *C. neoformans* was grown at 30 °C on Sabouraud dextrose agar (SDA; BD Biosciences). For liquid culture, the strains were incubated in an orbital shaker at 200 rpm.

### 4.3. Microbroth Dilution Assay

The antifungal activity of berberine was evaluated using a microbroth dilution assay, following the protocol described by Song et al. [41] and based on CLSI guidelines (M38-A) [42]. In summary, berberine was dissolved in DMSO to prepare a 4 mg/mL stock solution, while fluconazole (FLC), used as a positive control, was dissolved in DMSO at 1 mg/mL. Both solutions were stored at 4 °C for future use. Cultures of the fungal strains, with an OD_600_ between 0.03 and 0.06, were grown to the exponential phase. These were then diluted 1:10 in broth, except for *C. neoformans*, which was used directly without dilution. A volume of 195 µL of each culture was transferred into 96-well plates. Incubation times were 24 h for *C. albicans* and 48 h for *C. neoformans*. Inhibition was determined by subtracting the absorbance of the blank wells and then dividing the result by the average absorbance of the DMSO-only wells and multiplying by 100 to calculate the percentage inhibition.

For the filamentous fungus *T. mentagrophytes*, antifungal activity was assessed after culturing the fungus on SDA at 30 °C for two weeks to obtain conidia. A mixed suspension of conidia and hyphal fragments was prepared by covering the fungal colonies with sterile saline (0.85%) and gently rubbing them with an inoculation loop. The suspension was filtered through sterile lens paper to remove hyphal fragments, followed by centrifugation at 1000× *g* for 10 min to isolate the conidia. The conidia were washed twice in sterile saline and resuspended in RPMI 1640 medium (pH 7.0) supplemented with L-glutamine and 2% glucose. Using a hemocytometer, the concentration of conidia was adjusted to 1 × 10⁴ cells/mL.

For the antifungal susceptibility assay, the conidia suspension (190 μL) was added to 96-well plates containing a serial two-fold dilution of berberine (10 μL). Fluconazole at 20 μg/mL served as the positive control, while DMSO-only wells were used as the negative control. Plates were incubated at 30 °C for 72 h on a plate shaker, and the optical density (OD) at 510 nm was measured using a microplate reader (Bio-Rad, Hercules, CA, USA). The median inhibitory concentration was determined using GraphPad Prism 5, and the minimum inhibitory concentration (MIC) was defined as the concentration of berberine that reduced absorbance by at least 95% compared to the DMSO control.

### 4.4. Scanning Electron Microscopy (SEM)

Samples from both the control and berberine treatments (10 and 20 µM) were collected for SEM observation of cell morphology. After 24 h of incubation, the cells were fixed with 2.5% glutaraldehyde for 2–4 h. The samples were then sequentially transferred to alcohol solutions with increasing concentrations of 30%, 50%, 70%, and 90%, for 15 min each. Following this, the slides were coated with gold and observed under SEM at magnifications of 1000×, 5000×, and 10,000× (Thermo APREO S, Thermo Fisher Scientific, Waltham, MA, USA).

### 4.5. Transmission Electron Microscopy (TEM)

*C. neoformans* cells, at a concentration of 1 × 10^5^ CFU/mL, were incubated with either ketoconazole at a concentration of 10 µM or berberine at concentrations of 10 and 20 µM at a temperature of 30 °C. Following a 24-h period, the cells were collected, rinsed twice using PBS, and fixed in a solution of 2.5% glutaraldehyde overnight. Subsequently, they were treated with 1% osmium tetroxide (OsO_4_) dissolved in 0.1 M sodium cacodylate buffer at a pH of 7.4 for 1 h. The samples underwent a dehydration process using a series of acetone concentrations and were then embedded in EPON-812 resin (Merck, Rahway, NJ, USA). The ultrathin sections obtained were stained with a combination of 5% uranyl acetate and 5% lead citrate, after which they were observed using a transmission electron microscope (JEOL F200, Tokyo, Japan).

### 4.6. Flow Cytometry Assay

In this experiment, the Annexin V FITC/PI double staining method of flow cytometry was used to preliminarily determine the mode of action of the drug on fungi. Stationary-phase *C. neoformans* cells were refreshed and adjusted to 1 × 10^6^ CFU/mL in YM medium and subsequently cultured for 4 h with berberine at concentrations of 0.5 × MIC, 1 × MIC, and 2 × MIC. Afterward, the cells were prepared and analyzed using flow cytometry with a flow cytometry analyzer (Agilent, Palo Alto, CA, USA), following established protocols [14].

### 4.7. Fungal Intracellular Reactive Oxygen Species (ROS) Assay

The activated fungal suspension was collected and adjusted to a concentration of 1 × 10⁶ CFU/mL. DCFH-DA was added at a final concentration of 10 μM, followed by gentle mixing. The suspension was incubated for 30 min at 37 °C in a light-protected incubator, with shaking every 5 min. After incubation, the DCFH-DA-loaded fungal cells were washed three times with fresh medium. For *C. neoformans*, different concentrations of berberine were applied to the treatment groups, with 10 μM ketoconazole serving as a positive control. The control group received no treatment, and three replicates were set for each group. ROS signals were measured using flow cytometry in the FITC channel.

### 4.8. Mitochondrial Membrane Potential Assay

Rhodamine 123 was prepared into a 1 mM masterbatch for spare parts, and the activated organisms were diluted to 1 × 10^6^ CFU/mL; different concentrations for the dosing group and control group were set up, and a final concentration of 1–2 μM Rhodamine 123 was added to each group, and the incubation was protected from light for 30 min; different concentrations of berberine were added, and a normal control with no dosing and 10 μM ketoconazole was set up as the positive control, incubated for 6 h away from light; after collection, the organisms were washed with PBS 2~3 times, and fluorescence trends were observed with a flow cytometer in FITC channel.

### 4.9. In Silico Docking

Molecular docking studies were carried out using AutoDock Vina 1.1.1 (Scripps Research Institute, San Diego, CA, USA) to evaluate the binding affinity of various compounds. The crystal structure of CYP51 from *C. albicans* (Lanosterol 14-alpha demethylase) was retrieved from the Protein Data Bank (PDB ID: 5FSA). A docking grid of 75 × 75 × 70 points along the x, y, and z axes was defined, centering on the active site of posaconazole. The docking results, including the complex with the lowest binding energy and the binding interactions, were visualized using PyMOL Molecular Graphics System Version 1.3 (Schrödinger, LLC, Boston, MA, USA).

### 4.10. Binding Affinity Assay

The recombinant CYP51 from *C. albicans* was prepared at a concentration of 0.52 mg/mL, and the gradient concentrations of the test drug were prepared by serial dilution in PBS. Prior to the experiment, the APS sensor was equilibrated in PBS for 10 min. The initial concentration of berberine was set at 1 mM, followed by serial dilutions by a factor of 100 across five concentration levels. The experimental procedure was divided into five steps: baseline equilibrium, sample loading, equilibration, drug binding, and separation, with appropriate time intervals designated for each step. The probe was manually loaded onto a small-molecule affinity analyzer to execute the procedure. Baseline equilibrium was established for 30 s, followed by 240 s of protein sampling. The system was equilibrated and eluted with PBS for 90 s, after which the drug binding phase was carried out for 240 s, followed by an elution step of 240 s. The data collected after the baseline equilibrium phase were exported, and a time versus binding signal (nm) plot was generated.

### 4.11. Guinea Pig Dermatophytosis Model and Antifungal Treatment

A dermatophytosis model in guinea pigs was developed based on the protocol established by Song et al. [43]. *T. mentagrophytes* isolates were cultured on Sabouraud dextrose agar (SDA) plates at 30 °C for 14 days. Conidia were harvested and suspended at a concentration of 1 × 10^8^ conidia/mL, following standard preparation procedures. Briefly, before inoculation, female guinea pigs were anesthetized via intraperitoneal injection of chloral hydrate. The dorsal area was clipped and shaved, and two distinct 2.5 cm × 2.5 cm (6.25 cm^2^) sections were gently abraded using sterile fine-grit sandpaper. The conidial suspension (1 × 10^8^ conidia/mL in saline) was applied to the abraded skin and thoroughly rubbed in. To ensure successful establishment of the infection, skin scrapings from the inoculation sites were collected and examined microscopically using a KOH wet mount to identify hyphal structures. Ten days post-infection, 100 μL of either berberine or ketoconazole (used as a positive control), both at 10 mg/mL in saline, was applied topically to the infected areas once daily, equivalent to a dose of 1 mg/cm^2^. This treatment regimen continued for 10 days. The model group infected with *T. mentagrophytes* received normal saline, while the negative control group, which was not infected, was also treated with saline.

Following the treatment period, the skin of the guinea pigs was evaluated both morphologically and mycologically. Biopsy samples were taken under anesthesia from the treated areas after the 10-day treatment, immediately fixed in Carnoy’s solution within 24 h, sectioned at a thickness of 5 µm, and stained with PASM (periodic acid–silver methenamine) staining for histological examination under a light microscope.

### 4.12. Murine Model of C. neoformans Infection

Six-week-old female ICR mice were immunosuppressed via intraperitoneal (i.p.) injection of CY (200 mg/kg body weight; Meilun, Dalian, China) for the first 3 days. On Day 4, mice were inoculated i.p. with 200 µL of *C. neoformans* at a concentration of 1 × 10^8^ cells/mL in normal saline and then randomly assigned to five groups: model group, 5 mg/kg FLC group, 5 mg/kg berberine group, 10 mg/kg berberine group, and 15 mg/kg berberine group, with 10 mice per group. An additional group of 10 uninfected mice served as the normal control. One hour post-infection, each group received the assigned treatment or water via oral gavage, which was continued daily for 7 days. Body weight and survival rates were monitored over a 10-day period.

### 4.13. Histopathological Assay

On Day 10, mice were euthanized for lung tissue collection. In the model group, lung tissues from deceased mice were immediately harvested for histological analysis. The tissues were fixed in 4% (*w*/*v*) formaldehyde solution and then trimmed, washed, dehydrated, and embedded in paraffin, following the procedure described by Song et al. [44]. The samples were subsequently sectioned and stained with hematoxylin and eosin (H&E) for microscopic examination.

### 4.14. Statistical Analyses

Data are presented as the mean ± SD from three independent experiments. Statistical comparisons were made using one-way analysis of variance (ANOVA) followed by Tukey’s post hoc test. Data analysis was performed using SPSS software (version 17.0; SPSS Inc., Chicago, IL, USA), with statistical significance defined as *p* * < 0.05.

## 5. Conclusions

In conclusion, our study confirms and extends the antifungal potential of berberine, particularly against dermatophytes and other filamentous fungi, in addition to yeast-like fungi. The findings suggest that berberine could play an important role in the future management of fungal infections, including those caused by drug-resistant strains. Future research should focus on clinical applications, combination therapies, and a deeper understanding of berberine’s pharmacological mechanisms to fully harness its therapeutic potential in antifungal therapy.

## Data Availability

The original contributions presented in the study are included in the article, further inquiries can be directed to the corresponding authors.

## References

[1] Wei Y., Xu X.Y., Song X. (2017). A review of antifungal natural products against the pathogenic fungi causing athletes’ foot disease. Current. Organic. Chem..

[2] Zhang C.W., Zhong X.J., Zhao Y.S., Rajoka M.S.R., Hashmi M.H., Zhai P., Song X. (2023). Antifungal natural products and their derivatives: A review of their activity and mechanism of actions. Pharmacol. Res. Mod. Chin. Med..

[3] Kanafani Z.A., Perfect J.R. (2008). Resistance to antifungal agents: Mechanisms and clinical impact. Clin. Infect. Dis..

[4] Zhou H.A.O., Wang W.A.O., Cai L.A.O., Yang T.A.O. (2023). Potentiation and mechanism of berberine as an antibiotic adjuvant against multidrug-resistant bacteria. Infect. Drug Resist..

[5] Wang J., Wang L., Lou G.H., Zeng H.R., Hu J., Huang Q.W., Peng W., Yang X.B. (2019). Coptidis Rhizoma: A comprehensive review of its traditional uses, botany, phytochemistry, pharmacology and toxicology. Pharm. Biol..

[6] Ryuk J.A., Zheng M.S., Lee M.Y., Seo C.S., Li Y., Lee S.H., Moon D.C., Lee H.W., Lee J.H., Park J.Y. (2012). Discrimination of *Phellodendron amurense* and *P*. chinense based on DNA analysis and the simultaneous analysis of alkaloids. Arch. Pharm. Res..

[7] Gawel K., Kukula-Koch W., Nieoczym D., Stepnik K., Ent W.V., Banono N.S., Tarabasz D., Turski W.A., Esguerra C.V. (2020). The influence of palmatine isolated from berberis sibiricaradix on pentylenetetrazole-induced seizures in zebrafish. Cells.

[8] Neag M.A., Mocan A., Echeverría J., Pop R.M., Bocsan C.I., Crişan G., Buzoianu A.D. (2018). Berberine: Botanical occurrence, traditional uses, extraction methods, and relevance in cardiovascular, metabolic, hepatic, and renal disorders. Front. Pharmacol..

[9] Han Y., Lee J.H. (2005). Berberine synergy with amphotericin B against disseminated candidiasis in mice. Biol. Pharm. Bull..

[10] da Silva A.R., de Andrade Neto J.B., da Silva C.R., Campos R.D.S., Costa Silva R.A., Freitas D.D., do Nascimento F.B., de Andrade L.N., Sampaio L.S., Grangeiro T.B. (2016). Berberine antifungal activity in fluconazole-resistant pathogenic yeasts: Action mechanism evaluated by flow cytometry and biofilm growth inhibition in *Candida spp*. Antimicrob. Agents. Chemother..

[11] Zhang L., Wu X., Yang R., Chen F., Liao Y., Zhu Z., Wu Z., Sun X., Wang L. (2021). Effects of berberine on the gastrointestinal microbiota. Front. Cell. Infect. Microbiol..

[12] Harrison S.A., Gunn N., Neff G.W., Kohli A., Liu L., Flyer A., Goldkind L., Di A.M. (2021). A phase 2, proof of concept, randomised controlled trial of berberine ursodeoxycholate in patients with presumed non-alcoholic steatohepatitis and type 2 diabetes. Nat. Commun..

[13] Kosalec I., Jazvinšćak Jembrek M., Vlainić J. (2022). The spectrum of berberine antibacterial and antifungal activities. Promising Antimicrobials from Natural Products.

[14] Hu Z., Hu H., Hu Z., Zhong X., Guan Y., Zhao Y., Wang L., Ye L., Ming L., Riaz Rajoka M.S. (2022). Sanguinarine, isolated from macleaya cordata, exhibits potent antifungal efficacy against Candida albicans through inhibiting ergosterol synthesis. Front. Microbiol..

[15] Iyer K.R., Revie N.M., Fu C., Robbins N., Cowen L.A.O. (2021). Treatment strategies for cryptococcal infection: Challenges, advances and future outlook. Nat. Rev. Microbiol..

[16] Bermas A., Geddes-McAlister J.A.O. (2020). Combatting the evolution of antifungal resistance in *Cryptococcus neoformans*. Mol. Microbiol..

[17] Sousa N.A.O., Almeida J.A.O., Frickmann H.A.O., Lacerda M.A.O., Souza J.A.O. (2023). Searching for new antifungals for the treatment of cryptococcosis. Rev. Soc. Bras. Med. Trop..

[18] Wong-Deyrup S.W., Song X., Ng T.W., Liu X.B., Zeng J.G., Qing Z.X., Deyrup S.T., He Z.D., Zhang H.J. (2021). Plant-derived isoquinoline alkaloids that target ergosterol biosynthesis discovered by using a novel antifungal screening tool. Biomed. Pharmacother..

[19] Qin Z., Tang R., Liang J., Jia X. (2024). Berberine, a natural alkaloid: Advances in its pharmacological effects and mechanisms in the treatment of autoimmune diseases. Int. Immunopharmacol..

[20] Ye Y., Liu X., Wu N., Han Y., Wang J., Yu Y., Chen Q. (2021). Efficacy and safety of berberine alone for several metabolic disorders: A systematic review and meta-analysis of randomized clinical trials. Front. Pharmacol..

[21] Huang X., Zheng D., Yong J., Li Y. (2022). Antifungal activity and potential mechanism of berberine hydrochloride against fluconazole-resistant *Candida albicans*. J. Med. Microbiol..

[22] Huang X., Zheng M., Yi Y., Patel A., Song Z., Li Y. (2020). Inhibition of berberine hydrochloride on *Candida. albicans.* biofilm formation. Biotechnol. Lett..

[23] Yang F., Pei R., Zhang Z., Liao J., Yu W., Qiao N., Han Q., Li Y., Hu L., Guo J. (2019). Copper induces oxidative stress and apoptosis through mitochondria-mediated pathway in chicken hepatocytes. Toxicol. Vitr. Int. J. Publ. Assoc. BIBRA.

[24] Qian Z., Mengxun Z., Yingchao W., Tingting Z., Roujuan W., Shuhong W., Yi D., Ruirui Y., Peng Y., Yifan S. (2022). Natural compound 2-Chloro-1,3-dimethoxy-5-methylbenzene, isolated from Hericium. erinaceus, inhibits fungal growth by disrupting membranes and triggering apoptosis. J. Agric. Food Chem..

[25] Rizwan H., Pal S., Sabnam S., Pal A. (2020). High glucose augments ROS generation regulates mitochondrial dysfunction and apoptosis via stress signalling cascades in keratinocytes. Life Sci..

[26] Menzies F.M., Cookson M.R., Taylor R.W., Turnbull D.M., Chrzanowska-Lightowlers Z.M., Dong L., Figlewicz D.A., Shaw P.J. (2002). Mitochondrial dysfunction in a cell culture model of familial amyotrophic lateral sclerosis. Brain J. Neurol..

[27] Zhang J., Li L., Lv Q., Yan L., Wang Y., Jiang Y. (2019). The Fungal CYP51s: Their functions, structures, related drug resistance, and inhibitors. Front. Microbiol..

[28] Rosam K., Monk B.C., Lackner M. (2020). Sterol 14α-demethylase ligand-binding pocket-mediated acquired and intrinsic azole resistance in fungal pathogens. J. Fungi..

[29] Jordá T., Puig S. (2020). Regulation of ergosterol biosynthesis in Saccharomyces cerevisiae. Genes.

[30] Xie Y., Liu X., Zhou P. (2020). In vitro Antifungal Effects of Berberine against Candida spp. in planktonic and biofilm conditions. Drug Des. Dev. Ther..

[31] Nataša Z., Ivan K., Siniša T., Ivan B., Mario J., Toni V.l., Josipa V. (2017). Membrane of *Candida albicans* as a target of berberine. BMC Complement. Altern. Med..

[32] Luo X.F., Zhou H., Deng P., Zhang S.Y., Wang Y.R., Ding Y.Y., Wang G.H., Zhang Z.J., Wu Z.R., Liu Y.Q. (2024). Current development and structure-activity relationship study of berberine derivatives. Bioorganic. Med. Chem..

[33] Almatroodi S.A., Alsahli M.A., Rahmani A.H. (2022). Berberine: An important emphasis on its anticancer effects through modulation of various cell signaling pathways. Molecules.

[34] Luo H., Pan K.S., Luo X.L., Zheng D.Y., Andrianopoulos A., Wen L.M., Zheng Y.Q., Guo J., Huang C.Y., Li X.Y. (2019). In vitro susceptibility of berberine combined with antifungal agents against the yeast form of Talaromyces marneffei. Mycopathologia.

[35] Shao J., Shi G., Wang T., Wu D., Wang C. (2016). Antiproliferation of berberine in combination with fluconazole from the perspectives of reactive oxygen species, ergosterol and drug efflux in a fluconazole-resistant Candida tropicalis isolate. Front. Microbiol..

[36] Sajeev A., Sailo B., Unnikrishnan J., Talukdar A., Alqahtani M.S., Abbas M., Alqahtani A., Sethi G., Kunnumakkara A.B. (2024). Unlocking the potential of berberine: Advancing cancer therapy through chemosensitization and combination treatments. Cancer Lett..

[37] Li X., Song Y., Wang L., Kang G., Wang P., Yin H., Huang H. (2021). A potential combination therapy of berberine hydrochloride with antibiotics against multidrug-resistant Acinetobacter baumannii. Front. Cell. Infect. Microbiol..

[38] Wei G.X., Xu X., Wu C.D. (2011). In vitro synergism between berberine and miconazole against planktonic and biofilm Candida cultures. Arch. Oral. Biol..

[39] Simões Gobbi L.P., Costa E.H.E., Fernandez C.M.M., Lorenzetti F.B., Fonseca D.P., Gomes A.V., Baldoqui D.C., Fernandes C.S., Ueda-Nakamura T., Nakamura C.V. (2023). Berberine-fluconazole microparticle-based combination therapy to treat candidiasis infections. J. Appl. Microbiol..

[40] Iwazaki R.S., Endo E.H., Ueda-Nakamura T., Nakamura C.V., Garcia L.B., Filho B.P.D. (2010). In vitro antifungal activity of the berberine and its synergism with fluconazole. Antonie Van Leeuwenhoek.

[41] Song X., Gaascht F., Schmidt-Dannert C., Salomon C.E. (2020). Discovery of antifungal and biofilm preventative compounds from mycelial cultures of a unique North American Hericium sp. fungus. Molecules.

[42] Clinical and Laboratory Standards Institute (2018). Performance Standards for Antimicrobial Disk Susceptibility Tests.

[43] Song X., Wei Y.X., Lai K.-M., He Z.D., Zhang H.J. (2018). In vivo antifungal activity of dipyrithione against Trichophyton rubrum on guinea pig dermatophytosis models. Biomed. Pharmacother..

[44] Song X., He J., Xu H., Hu X.P., Wu X.L., Wu H.Q., Liu L.Z., Liao C.H., Zeng Y., Li Y. (2016). The antiviral effects of acteoside and the underlying IFN-γ-inducing action. Food Funct..

